# Perfoliate Pondweed Meadows in Northern Coastal Areas—Reservoirs of Diverse Bacteria With Pathogenic Traits and Complex Carbon Metabolism

**DOI:** 10.1111/1758-2229.70180

**Published:** 2025-09-12

**Authors:** Kesava Priyan Ramasamy, Máté Vass, Johnny Berglund, Anniina Saarinen, Agneta Andersson

**Affiliations:** ^1^ Department of Ecology and Environmental Science Umeå University Umeå Sweden; ^2^ Umeå Marine Sciences Centre Umeå University Umeå Sweden; ^3^ Division of Systems and Synthetic Biology, Department of Life Sciences, Science for Life Laboratory Chalmers University of Technology Gothenburg Sweden; ^4^ County Administrative Board of Västerbotten Umeå Sweden

**Keywords:** 16S rRNA metabarcoding, epiphytes, metabolic pathways, *Potamogeton perfoliatus*, potential pathogens

## Abstract

The perfoliate pondweed, *Potamogeton perfoliatus* , is a common macrophyte in freshwater and subarctic coastal areas. This species builds extensive meadows that play a role as a filter removing nutrients traversing from land to sea and maintain essential ecosystem functions. Here, we investigated the function of perfoliate pondweed as a filter of potentially pathogenic bacteria by combining culture‐dependent and 16S rRNA metabarcoding approaches. Our results suggest no significant nutrient reduction in the meadow region but the enrichment of potentially pathogenic bacteria, such as *Vibrio*, *Legionella* and *Leptospira,* particularly attached to macrophyte leaves. The bacterial community composition differed between seawater and macrophyte habitats, with higher relative abundances of Cyanobacteriia attached to macrophytes, without affecting alpha‐diversity. The metabolic pathways of bacteria for aromatic and polymer compound degradation were enriched in the macrophytes, attributed to members of the genera *Pseudorhodobacter*, *Novosphingobium* and *Erythrobacter*. Functions related to such degradation suggest that the bacteria may be able to remove complex organic compounds coming from land. We argue that the macrophyte meadows may be relevant to both animal and human health, as these habitats can be hot spots for potentially pathogenic bacteria.

## Graphical abstract

The perfoliate pondweed, *Potamogeton perfoliatus* , is a common macrophyte in freshwater and subarctic coastal areas. This species builds extensive meadows that play a role as a filter removing nutrients traversing from land to sea and maintaining essential ecosystem functions. We investigated the function of perfoliate pondweed as a filter of potentially pathogenic bacteria by combining culture‐dependent and 16S rRNA metabarcoding approaches.

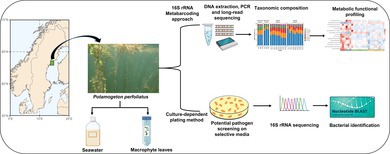

## 1 Introduction

Macrophyte meadows are widespread in shallow lakes and coastal areas, and they have a functional role in filtering nutrients that are traversing from land to aquatic systems (Tang et al. 2017; Brisson et al. 2020). Studies have shown that macrophyte communities can be used as indicators of water quality in aquatic habitats (Takamura et al. 2003; Søndergaard et al. 2010; Hansen and Snickars 2014; Poikane et al. 2018; Rajala et al. 2024). In many aquatic systems, the macrophyte diversity has decreased over the past years due to environmental change, such as eutrophication, land use and climate change (Sand‐Jensen et al. 2000; Steffen et al. 2013; Puche et al. 2024; Li et al. 2017; Lammerant et al. 2024), implying that certain ecosystem functions might be impaired.

The meadows provide shelter and habitat texture for many organisms and they are of outermost importance for the recruitment of many fishes (Hansen et al. 2019). Macrophytes harbour diverse epiphytic communities and are crucial for nutrient cycling (Hempel et al. 2008; Mentes et al. 2017; Chao et al. 2021; Koehl 2022). The surfaces of leaves, roots, rhizomes and thalli are habitats with tight relationships between microbes and macrophytes (Korlević et al. 2021). Such compartments on macrophytes provide optimal conditions for bacterial growth and metabolism, for example enabling access to dissolved organic carbon (DOC), oxygen and macronutrients (Sand‐Jensen et al. 1982; Carignan and Kalff 1982; Theil‐Nielsen and Sondergaard 1999; Hollants et al. 2013). Because of the proximity of different types of organisms inhabiting the meadows, there might be significant dispersal of fish microorganisms to macrophytes and vice versa.

Certain rhizospheric bacteria associated with macrophytes have been shown to be capable of degradation of aromatic carbons, such as phenols, which may make them usable for remediation purposes (Toyama et al. 2009; Yan et al. 2022). The ecological impact is, however, not known. A recent study showed relatively high abundances of Proteobacteria and Bacteroidota growing on macrophytes, and that the functions for carbon metabolism were enriched compared to that in seawater bacteria (Chen et al. 2022). In the boreal region, huge amounts of terrestrial organic matter are transported from land to sea (Gustafsson et al. 2014), which causes high DOC concentrations and humic aromatic substances in lakes, rivers and coastal regions, for example in the northern Baltic Sea (Skoog et al. 2011; Lønborg et al. 2024). As the macrophyte meadows are widespread, epiphytic microorganisms, with the capabilities of degrading various organic compounds, may have a role as degraders of terrestrial organic matter traversing from land to sea. Therefore, it is important to elucidate if enrichment of carbon metabolic functions in epiphytic bacteria is a widespread phenomenon.

Some studies have reported that macrophytes are reservoirs of bacterial pathogens, such as *Aeromonas*, Enterobacteriaceae, and *Vibrio* (Mathai et al. 2019; Reusch et al. 2021; Kalvaitienė et al. 2023; Gebbe et al. 2024). There is, however, no general consensus about this phenomenon, as other studies have evidenced higher abundances of potential pathogenic bacteria in seawater than those attached to macrophytes (Chen et al. 2022). Seagrass meadows of marine environments have been shown to reduce the abundance of potentially pathogenic bacteria from the surrounding seawater, which otherwise may cause disease in humans and marine organisms (Lamb et al. 2017; Quero et al. 2015). The macrophytes might thus have a filtering role when water moves from land to sea. Alternatively, the macrophyte meadows may also serve as a reservoir of potentially pathogenic bacteria, as they constitute important habitats for fish and other organisms that can carry pathogenic bacteria.

The perfoliate pondweed, *Potamogeton perfoliatus* , is a submerged perennial aquatic plant found in various habitats of freshwater and brackish water, and a common macrophyte in the Baltic Sea (Andersson 2000). Hence, we studied the epiphytic bacterial community growing on this macrophyte and compared it with that in seawater in two nearby bays in the subarctic northern Baltic Sea. We would argue that if the macrophytes harbour potentially pathogenic bacteria, then the established view of meadows indicating good water quality may be re‐evaluated. Further, we wanted to elucidate if the meadows function as a filter of nutrients, macromolecules, and potentially pathogenic bacteria in the studied coastal area. By utilising the combination of long‐read metabarcoding and selective plating techniques of pathogens, we investigated if the macrophyte meadows reduce the nutrient concentrations and potentially pathogenic bacteria from inshore to offshore waters, and how the taxonomic and functional diversity varies among the seawater and epiphytic bacterial communities.

## 2 Materials and Methods

### 2.1 Sampling

**FIGURE 1:**
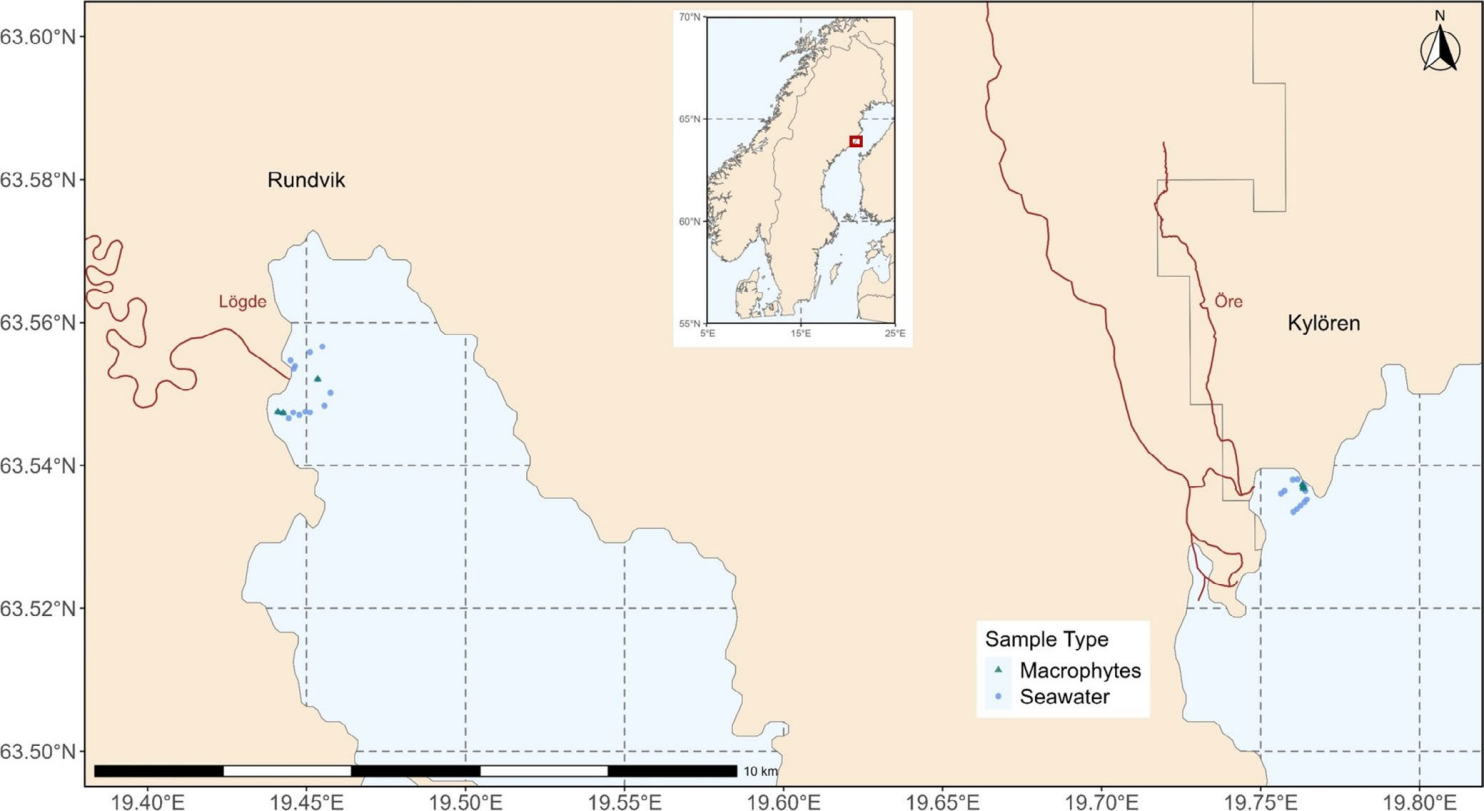
Sampling locations for macrophytes and seawater in the northern Baltic Sea.

In order to gain support for ecological patterns, two replicated bays with perfoliate macrophyte meadows were sampled in a coastal area in the northern Baltic Sea (Bothnian Sea), the Rundvik and Kylören Bays (Figure 1 and Table S1). The bays are situated 14 km apart and receive freshwater inflows via forest rivers. Specifically, the Lögde River (200 km long) enters the Rundvik Bay and the Öre river (225 km long) enters the Bothnian Sea close to the Kylören Bay. Seawater and macrophyte samples were collected in both bays in August 2022, during the peak growth season of *P. perfoliatus* (Santamaría 2002). Neither of the study areas is severely influenced by anthropogenic activity, as the population in Rundvik is low (800 persons) and in Kylören there are only a few summer houses. There is a municipal sewage treatment plant in Rundvik, comprising mechanical, chemical and biological cleaning of wastewater. In Kylören, there are only a couple of private households (~3) having 3‐chamber septic tanks in the nearshore area. The human impact on the bays should thus be relatively low.

In each bay, 15 seawater samples were collected at 0.5 m depth using a Ruttner sampler: five 1‐L samples from inshore, within and offshore the macrophyte meadows, respectively. The in situ temperature was recorded using a calibrated thermometer. Salinity ranged from < 0.5 to 4 PSU. For macrophyte sampling, one leaf from each of three *P. perfoliatus* plants (hereafter referred to as macrophytes) was randomly collected in the meadows and stored in 50 mL Falcon tubes containing 30 mL of 0.22 μm filtered seawater. All collected samples were immediately transported to the laboratory for further processing. These samples were shaken vigorously for 1 min to detach loosely attached epiphytic bacteria.

### 2.2 Chemical Analyses

Nutrient analysis was performed for all 30 seawater samples, measuring DOC, total dissolved phosphorus (TDP) and total dissolved nitrogen (TDN). Seawater was pre‐filtered using a 0.22 μm Supor membrane syringe filter (non‐pyrogenic; Acrodisc). DOC samples were acidified with HCl (18 mM final concentration). DOC was analysed on a Shimadzu (Kyoto) TOC‐5000 high‐combustion analyser, and TDN and TDP on a QuAAtro39 autoanalyser after an oxidation step using peroxodisulfate, according to standard analytical methods (Grasshoff et al. 2009). To test the differences in nutrient concentration between two bays, a Mann–Whitney *U* test was performed.

### 2.3 Bacterial Taxonomy and Metabolic Functions

#### 2.3.1 Sample Processing

Three of the seawater samples in each bay and sub‐habitat (inshore, within and offshore the meadows) and all three water samples containing the detached epiphytic bacteria from macrophyte leaves in each bay were filtered on sterile 0.22 μm filters (Pall Corporation sterilised filters, Supor, 47 mm, S‐pack white gridded) and stored in 1.5 mL Eppendorf tubes with 750 μL of DNA shield (Zymo Research, USA) at −80°C until processing for DNA extraction.

#### 2.3.2 DNA Extraction and Nanopore 16S rRNA Sequencing

DNA was extracted from the filtered samples using a ZymoBIOMICS DNA/RNA Miniprep Kit according to the manufacturer's instructions and quantified by using Qubit 1× HS Assay Kit (Invitrogen, Waltham, MA, USA). DNA was amplified by PCR using the 16S rRNA primers: 27F (5′‐AGRGTTYGATYMTGGCTCAG‐3′) (Lane 1991) and 1492R (5′‐RGYTACCTTGTTACGACTT‐3′) (Stackebrandt and Liesack 1993). The PCR conditions are as follows: an initial denaturation at 94°C for 3 min, followed by 35 cycles of denaturation at 94°C for 45 s, annealing at 50°C for 1 min, and extension at 72°C for 1.5 min, with a final extension at 72°C for 10 min. The PCR products were visualised in 1% agarose gel electrophoresis stained with SYBR green. The amplicons were purified with the AMPure magnetic beads (Beckmann) according to the manufacturer's instructions and quantified using Qubit 1× HS Assay Kit (ThermoFisher Scientific). The amplicon products were pooled in equimolar concentration and once again purified. The pooled library (1 μg DNA) was further processed according to the SQK‐LSK109 (Oxford Nanopore Technologies) kit, then sequenced on a MinION Mk1C instrument (ONT) with R9.4.1 flowcell. The basecalling was done using the High‐accuracy basecalling (HAC) mode using the MinKNOW software.

#### 2.3.3 Bioinformatics and Statistics

Quality reads were demultiplexed and barcoded primers were trimmed with MiniBar (Krehenwinkel et al. 2019); then filtered by length (1.2–1.8 kb) with NanoFilt (v2.8.0) (De Coster et al. 2018). These filtered quality reads were processed using the NGSpeciesID pipeline (v0.1.2.2) (Sahlin et al. 2021; Pomerantz et al. 2022), as described in Vass et al. (2022).

We obtained on average 100,830 reads per sample with a mapping rate (percent of high‐quality paired reads for generation of the consensus sequences from total reads) of 49.9% (Table S2). Quality‐filtered (*Q* > 9 and 1.2–1.8 kb) reads were deposited to NCBI SRA database under the accession number: PRJNA922946. The final, polished consensus sequences were used in a BLASTn search to assign taxonomy against the SILVA SSU v138.1 reference database (Quast et al. 2012) using BLAST+ (v2.11.0+) and keeping hits with at least 80% identity, as described in Vass et al. (2022). Results of the BLASTn search were processed with phyloR (https://github.com/cparsania/phyloR) to keep top hits and to assign taxonomy levels. Chloroplasts and mitochondria were excluded for further analysis. In the end, 604 OTUs were identified as bacteria; thus, they were kept for downstream analyses. The table was used to calculate the relative abundance of bacterial taxa. The OTU table (see Table S3) was processed using the vegan and phyloseq packages (Oksanen et al. 2022; McMurdie and Holmes 2013).

Significantly enriched bacterial genera on macrophyte leaves relative to those in seawater were identified using linear discriminant analysis (LDA) effect size (LEFSe) analysis in the microbiomeMarker package (Cao et al. 2022). To find a correlation between the occurrence of bacterial genera (both on macrophyte leaves and in seawater) and measured environmental variables, Pearson correlation analysis was performed using the R microeco package (v1.14.0) (Liu et al. 2021).

#### 2.3.4 Metabolic Functional Profiling and Diversity

Functional profiling based on 16S rRNA sequences was predicted using PICRUSt2 (Phylogenetic Investigation of Communities by Reconstruction of Unobserved States) software (Douglas et al. 2020). The abundance of KEGG Orthology (KO) pathways and the MetaCyc pathways from PICRUSt2 output files were analysed and visualised using the ggpicrust2 package (Yang et al. 2023). Alpha diversity (Shannon index, and species richness) was estimated using the R package ‘hillR’ (Li 2018). To test the statistical significance of alpha diversity measures between bays and sample types, the Wilcoxon rank–sum test was performed. To compare the bacterial composition between seawater and macrophyte meadows, non‐metric multidimensional scaling (NMDS) was performed using Bray–Curtis distance matrices calculated with the vegan R package (Oksanen et al. 2022) and plotted using the ggplot2 package (Wickham 2016) in R. Permutational multivariate analysis of variance (PERMANOVA) (Anderson 2017) was performed using the vegan R package. All analyses and plotting were performed using R software (v 4.3.0, R Core Team 2021).

### 2.4 Bacterial Isolation Using Selective Media

The occurrence of potentially pathogenic bacteria in all sampling locations (*n* = 24) was analysed using two selective media: Coliform ChromoSelect and thiosulfate citrate bile sucrose (TCBS) (Merck, Darmstadt, Germany) for the isolation of coliform and *Vibrio* species. Aliquots of 100 μL seawater and macrophyte rinsed filtered seawater samples were inoculated on agar plates using the spread plate method. Each sample was spread on agar plates in triplicates. The inoculated plates were incubated at 22°C for 3 days. The number of visible colonies on agar plates was counted to enumerate the colony forming unit (CFU) counts. After counting, randomly selected morphologically distinct colonies (*n* = 39) were streaked on agar plates to obtain individual pure cultures for 16S rRNA gene sequencing. Further, the colonies were stored at a final concentration of 25% glycerol at −80°C for long‐term preservation. ANOVA and Tukey's HSD post hoc tests were performed for CFU of bacteria for the comparison of CFU counts of bacteria among bays and sample types.

#### 2.4.1 DNA Extraction From Isolates and 16S rRNA Gene Sequencing

Prior to DNA extraction, to gain sufficient DNA material for subsequent processes, colonies from Coliform ChromoSelect agar plates were grown in 5 mL of lysogeny broth (LB) composed of tryptone 10 g/L, Yeast Extract 5 g/L, Sodium Chloride 10 g/L. Colonies from TCBS agar plates were grown in 5 mL of Alkaline Saline Peptone Water (ASPW) (Sigma–Aldrich). Genomic DNA of isolated bacteria was extracted with DNeasy UltraClean Microbial Kit (Qiagen) according to the manufacturer's instructions. The purity of the extracted DNA was analysed by spectrophotometry using an ND‐1000 spectrophotometer (NanoDrop Technologies, Wilmington, DE) and using 1× dsDNA High Sensitivity (HS) kit (Thermo Fisher Scientific) according to the manufacturer's instructions. For 16S rRNA gene amplification, we used a standard PCR protocol; the 16S rRNA region was amplified using 27F (5′‐AGAGTTTGATCMTGGCTCAG‐3′) and 1492R (5′‐TACGGYTACCTTGTTACGACTT‐3′) (Heuer et al. 1997). The PCR was performed in a total 50 μL using HotStar Taq Mastermix (Qiagen); PCR conditions were as follows: initial denaturation (95°C for 15 min), followed by 30 cycles of denaturation (95°C for 60 s), annealing (50°C for 45 s), extension (72°C for 90 s), and a final extension step at 72°C for 10 min. The PCR products were detected on a 1% agarose gel electrophoresis and further purified using the Qiaquick PCR purification kit (Qiagen) according to the manufacturer's instructions. Sequencing of the 16S rRNA gene was performed using Sanger sequencing at Eurofins Genomics, Germany. The sequence quality was checked using Bioedit 7.0 (Hall 1999) and contigs were generated. The 16S rRNA sequence similarity was determined using BLASTn against the Core Nucleotide (nt) database at the National Centre for Biotechnological Information (NCBI). The GenBank accession numbers for these isolate‐related 16S rRNA gene sequences have been deposited in GenBank under the accession numbers (OP616840‐OP616850 and OP616944‐OP616971).

## 3 Results

### 3.1 Physicochemical Variables in Inshore, Within and Offshore the Meadow Waters

The seawater temperature varied between 14°C and 16°C at all sampling locations (Table S1). In Rundvik Bay, the DOC and TDN concentrations were higher in the inshore water than in the meadow and offshore waters (Figure S1A,B), possibly due to the adjacent entrance of Lögde River. TDP showed a contrasting spatial pattern, with higher average values in the meadow and offshore water (Figure S1C). In Kylören Bay, the DOC, TDN and TDP concentrations were similar in inshore, within and offshore the macrophyte meadows (Figure S1A–C), as the river inflow is positioned further outside the bay. There were, however, no statistically significant differences in DOC concentrations between Rundvik and Kylören Bay (Mann–Whitney test, *p* > 0.01). Overall, TDN concentration was higher in Rundvik Bay (Mann–Whitney test, *p* < 0.01) than in Kylören Bay.

### 3.2 Bacterial Communities on Macrophytes and Surrounding Seawater

**FIGURE 2:**
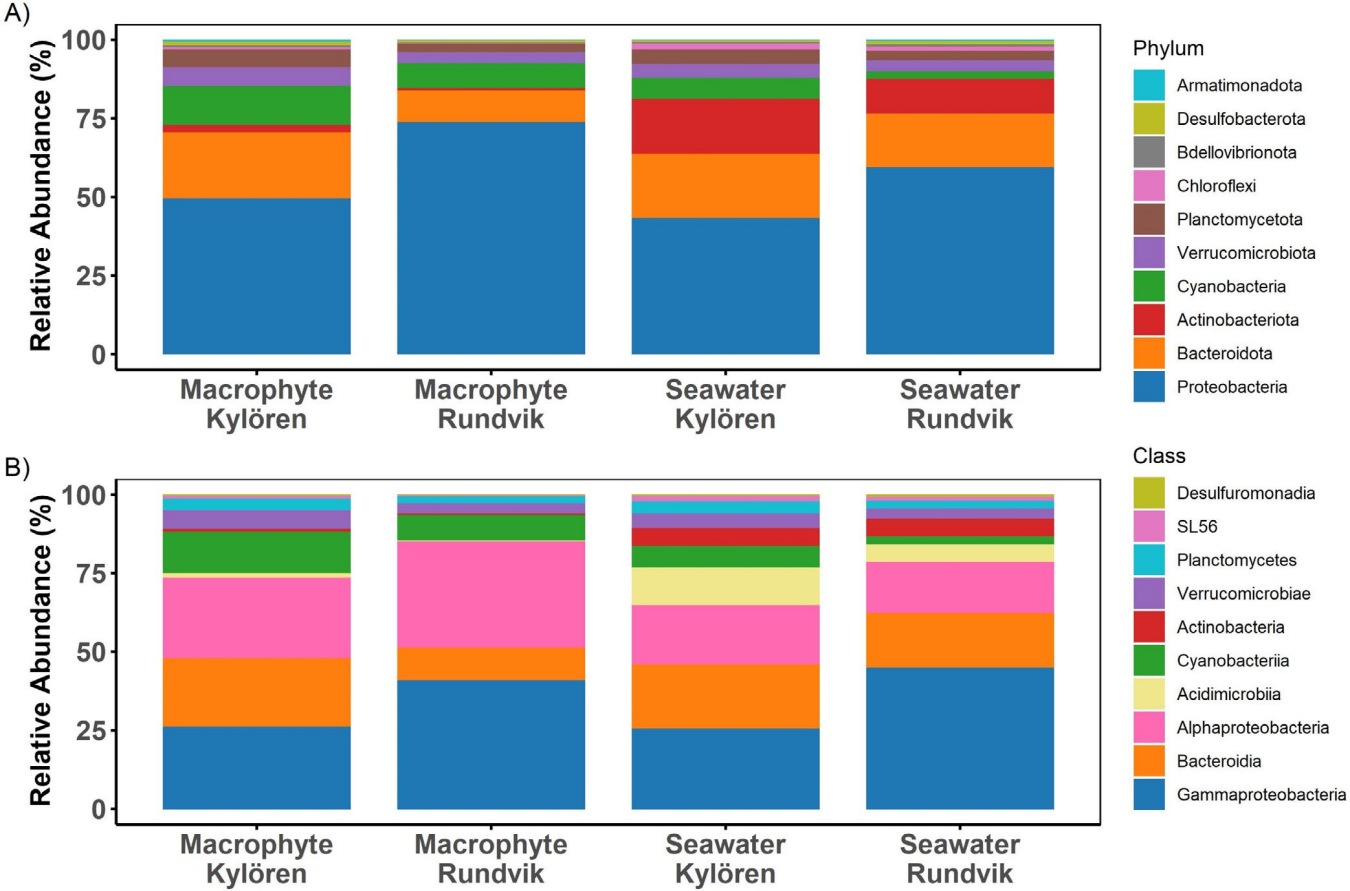
Distribution of bacterial communities based on the relative abundance of the top 10 (A) bacterial phyla and (B) bacterial classes associated with macrophytes and seawater in Rundvik and Kylören bays. Samples are grouped based on sample types: (macrophytes: epiphytes) and seawater (inshore, meadow region and offshore).

Metabarcoding showed that bacterial communities consisted of 21 phyla, where Proteobacteria, Bacteroidota, Actinobacteriota, Cyanobacteria, Verrucomicrobiota, Planctomycetota, Chloroflexi, Bdellovibrionota, Desulfobacterota and Armatimonadota constituted the top 10 most abundant ones (Figure S2 and Figure 2A). Proteobacteria was the dominant bacterial phylum in both habitat types, constituting 43%–74% of the community (Figure 2A). Cyanobacteria was more abundant on macrophytes than in seawater, constituting 8%–12% on macrophytes, whereas only 2%–7% of the community in seawater (Figure 2A). Actinobacteriota, on the other hand, was more abundant in seawater than on macrophytes, constituting 11%–18% in seawater and 1%–2% on macrophytes (Figure 2A). Some differences were also found between the bays, as for example, Bacteroidota that was somewhat more prominent in Kylören Bay in both habitat types (Figure 2A).

**FIGURE 3:**
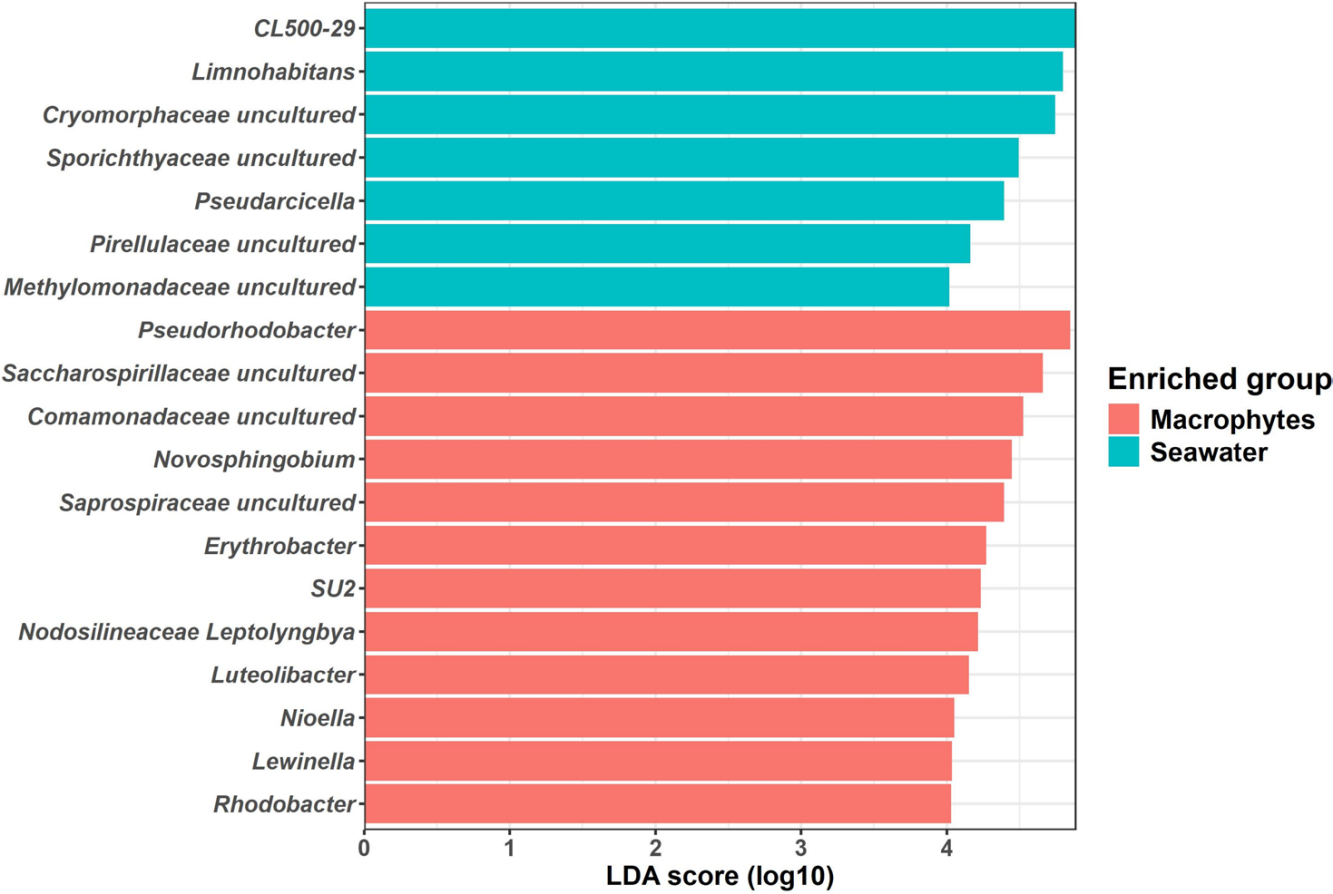
Bacterial genera that differ between macrophyte and seawater samples based on LEFSe analysis. Genera with the highest linear discriminant analysis (LDA) scores (Wilcoxon_cutoff = 0.05, LDA_cutoff = 4) are displayed.

At the class level, Gammaproteobacteria, Bacteroidia and Alphaproteobacteria were the most prevalent groups in both habitat types, together constituting > 70% of the bacterial communities (Figure 2B). Some differences were observed between the bays, as for example Gammaproteobacteria that was more abundant in Rundvik than in Kylören Bay. Notably, Alphaproteobacteria and Cyanobacteriia were more abundant on macrophytes than in seawater, while Acidiomicrobiia and Actinobacteria were more prominent in seawater (Figure 2B). The dominant families on macrophytes were Rhodobacteraceae (17%–34%) and Sphingomonadaceae (7%–19%), while the relative abundances of these families were lower in seawater samples (Figure S3). Notably, the family Spirosomoaceae was highest in seawater in Rundvik Bay (Figure S3). The genera *Pseudorhodobacter*, *Novosphingobium*, *Erythrobacter*, *Luteolibacter*, *Nioella*, *Lewinella* and *Rhodobacter* (*p* < 0.05) were significantly enriched on macrophytes compared to seawater in both bays. In contrast, CL500‐29, *Limnohabitans* and *Pseudarcicella* (*p* < 0.05) were enriched in seawater (Figure 3).

**FIGURE 4:**
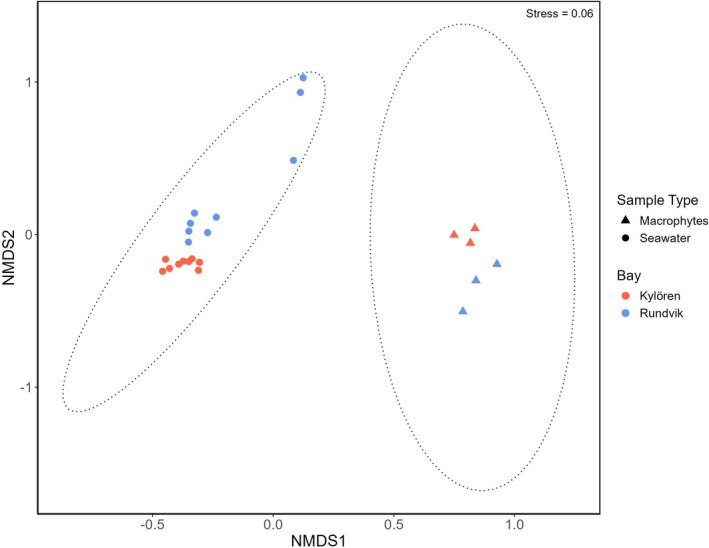
Non‐metric multidimensional scaling (NMDS) based on the relative abundance of OTUs using Bray–Curtis dissimilarity to visualise bacterial communities associated with macrophytes and seawater samples. Colours indicate samples collected from different bays, and symbols refer to sample types.

Taxa richness varied between 358 and 587 OTUs across all sampling locations (Figure S4A). The alpha diversity results suggest that there are no significant differences in taxa richness between macrophytes and seawater samples, whereas Shannon diversity was higher in macrophytes (*p* < 0.05, Wilcoxon rank–sum test; Figure S4B). The taxonomic composition, on the contrary, visualised by NMDS based on Bray–Curtis dissimilarity, showed that the bacterial community composition differed between seawater and macrophytes (ANOSIM: *R* = 0.829, *p* = 0.001; PERMANOVA: *F* = 37.89, *R* ^2^ = 0.632, *p* = 0.001, but was relatively similar in the two bays ANOSIM: *R* = 0.203, *p* = 0.008; PERMANOVA: *F* = 1.806, *R* ^2^ = 0.076, *p* = 0.211) (Figure 4).

### 3.3 Functional Metabolic Predictions

**FIGURE 5:**
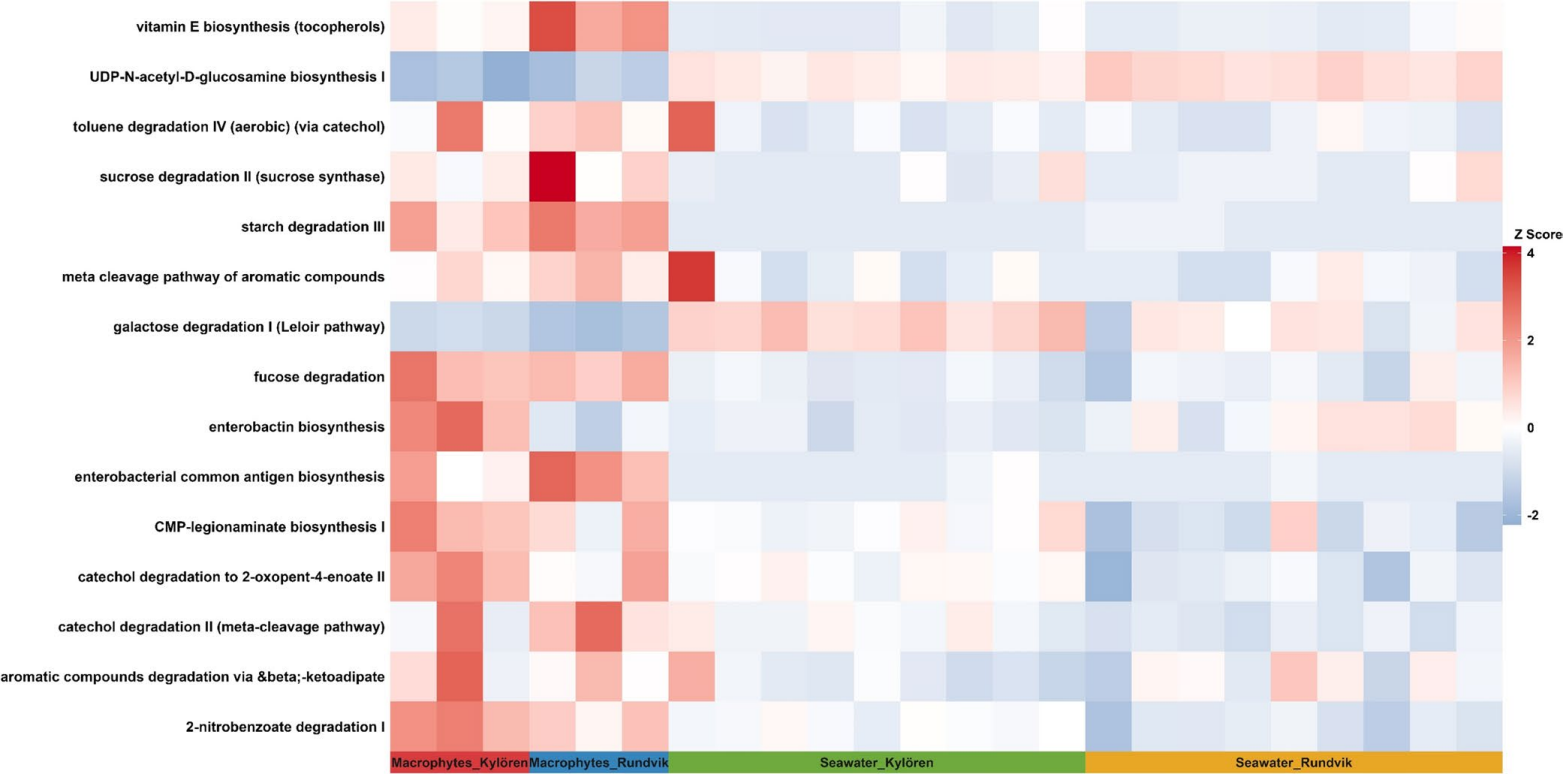
Heat map analysis showing the relative abundance of selected MetaCyc pathways involved in the bacterial metabolism of macrophyte‐associated samples compared with seawater. Features with *p* value < 0.05 identified using LinDA method (linear models for differential abundance analysis of microbiome compositional data) and *p* value adjusted using BH: Benjamini–Hochberg correction were considered significant.

The relative abundance of predicted metabolic pathways and KO features is summarised in Figure S5. In total, 14 pathways differed significantly (*p* value threshold = 0.05) between macrophyte and seawater samples. Specifically, on macrophytes, gene families related to cellular processes, cellular transport and catabolism, human diseases, metabolism, biosynthesis of secondary metabolites, glycan biosynthesis and amino acid metabolism were enriched (*p* < 0.05) compared to seawater samples. The analysis of MetaCyc pathways identified a total of 348 pathways across the samples (Table S4). Among these pathways, further analysis revealed those exhibiting the most significant differences (*p* < 0.05). The pathways for aromatic compounds such as meta cleavage of aromatic compounds, toluene degradation IV (aerobic) (via catechol), nitrobenzoate degradation and catechol degradation were enriched in epiphytic communities compared to seawater communities (Figure 5). The pathways associated with CMP‐legionaminate biosynthesis I and enterobacterial common antigen (ECA) biosynthesis were also enriched on macrophytes. The pathways for carbohydrate metabolism such as starch degradation III, fucose degradation and sucrose degradation were relatively higher in epiphytic communities compared to seawater communities. In contrast, pathways such as UDP‐N‐acetylglucosamine biosynthesis I and galactose degradation were more abundant in seawater samples (Figure 5).

### 3.4 Occurrence of Potentially Pathogenic Bacteria

**FIGURE 6:**
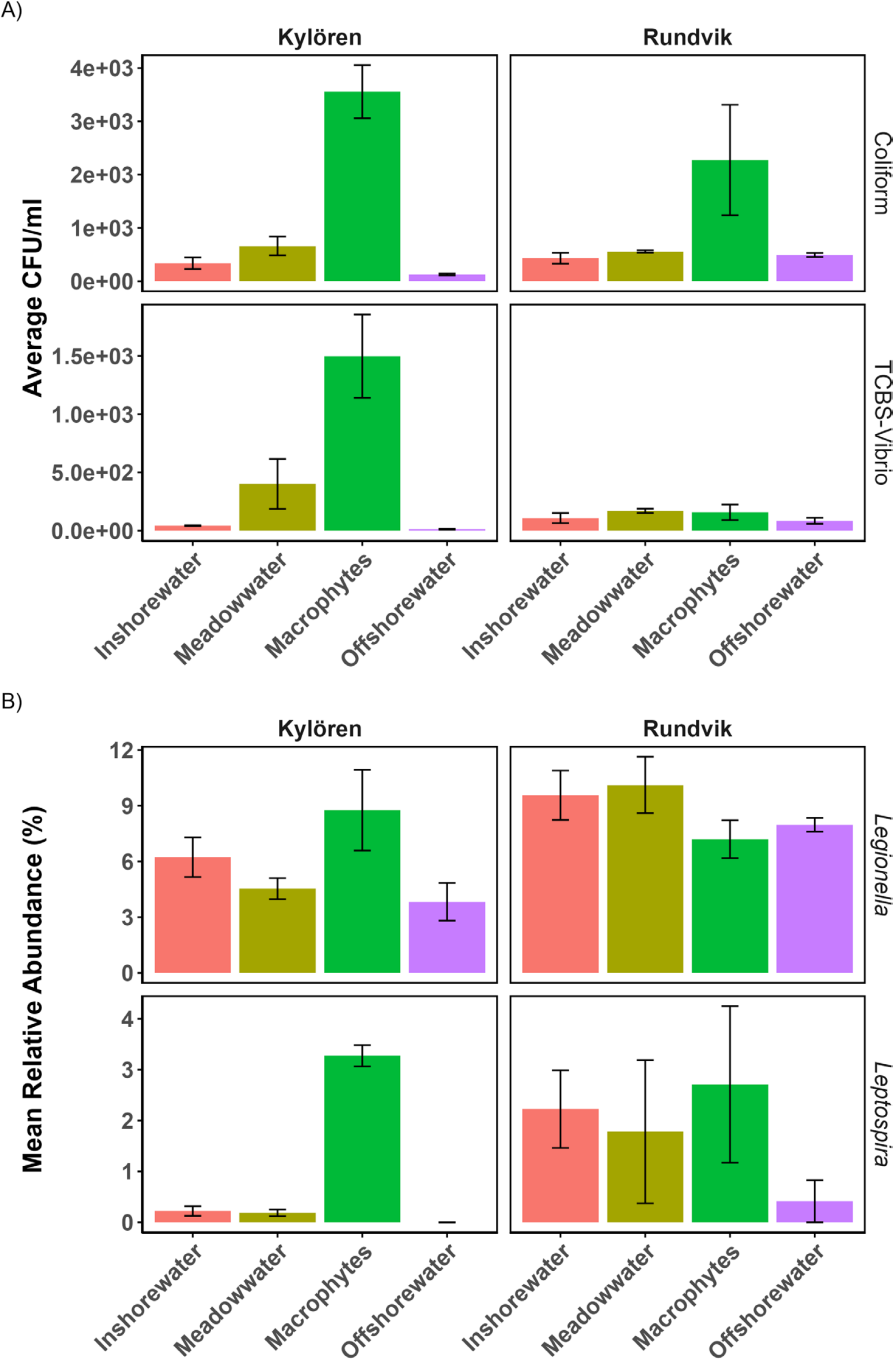
(A) Bacterial abundance (expressed as colony‐forming units, CFU) of selective pathogens in seawater and macrophytes of Kylören and Rundvik Bay. Error bar denotes standard error of three replicates. (B) Occurrence and mean relative abundance of two selected potentially pathogenic bacteria based on 16S rRNA metabarcoding data (*n* = 3 for macrophytes, *n* = 5 for seawater samples). Error bar represents standard error.

In both bays, higher abundances of CFU on ChromoSelect and TCBS agar consisted of coliforms and *Vibrio*‐related species that were observed among macrophyte‐associated bacteria than in seawater samples (Figure 6A). Additionally, based on random selection of colonies for 16S rRNA sequencing, we found some taxa of *Serratia* and *Aeromonas* were also prevalent on macrophytes. The bacterial counts on macrophyte samples were primarily coliforms, with an average CFU of 2.3 × 10^3^ CFU/mL in Rundvik Bay, which increased to an average of 3.5 × 10^3^ CFU/mL in Kylören Bay. In Kylören Bay, coliforms had significantly higher CFU counts as epiphytes compared to those in inshore (Tukey's post hoc, *p* < 0.001) and offshore seawater (Tukey's post hoc, *p* < 0.001). Comparison of CFU counts between seawater and macrophyte revealed significant differences with higher values among the epiphytic bacteria in the meadows (Tukey's post hoc, *p* < 0.001). Additionally, we found significant differences in the average CFU counts of bacterial colonies of *Vibrio* and other species grown on the TCBS media between Kylören and Rundvik Bays (Tukey's post hoc, *p* < 0.01). In Kylören Bay, the macrophyte had significantly higher abundances of both *Vibrio* and coliform bacteria than the offshore water. Additionally, in Kylören Bay, the inshore seawater had higher average abundances of both *Vibrio* and coliform bacteria than the offshore water. In Rundvik Bay, both meadow water and macrophytes showed higher CFU counts of coliform and *Vibrio* compared to inshore and offshore seawater (Figure 6A).

The metabarcoding data analysis revealed the occurrence of some potentially pathogenic bacteria within the genera *Leptospira* and *Legionella*. The average relative abundance of *Leptospira* was higher in the inshore of both bays (0.2% in Kylören and 2.2% in Rundvik Bay) compared to offshore seawater samples (not detected in Kylören and 0.4% in Rundvik Bay). Similarly, for *Legionella*, the relative abundance was higher in the inshore (6.2% in Kylören and 9.6% in Rundvik Bay) compared to offshore seawater (3.8% in Kylören and 8% in Rundvik Bay). In Kylören Bay, *Leptospira* OTUs showed higher relative abundance on the macrophyte (3.2%) than in the inshore seawater (0.2%) (Figure 6B). A consistent pattern of the average relative abundance was observed in both bays, although we found no statistically significant differences of these genera between inshore and offshore seawater samples (Tukey's post hoc, *p* > 0.05). However, the relative abundance of *Legionella* was significantly higher in Rundvik than in Kylören Bay (Tukey's post hoc, *p* < 0.01).

### 3.5 Relation Between Bacterial Genera and Environmental Variables

**FIGURE 7:**
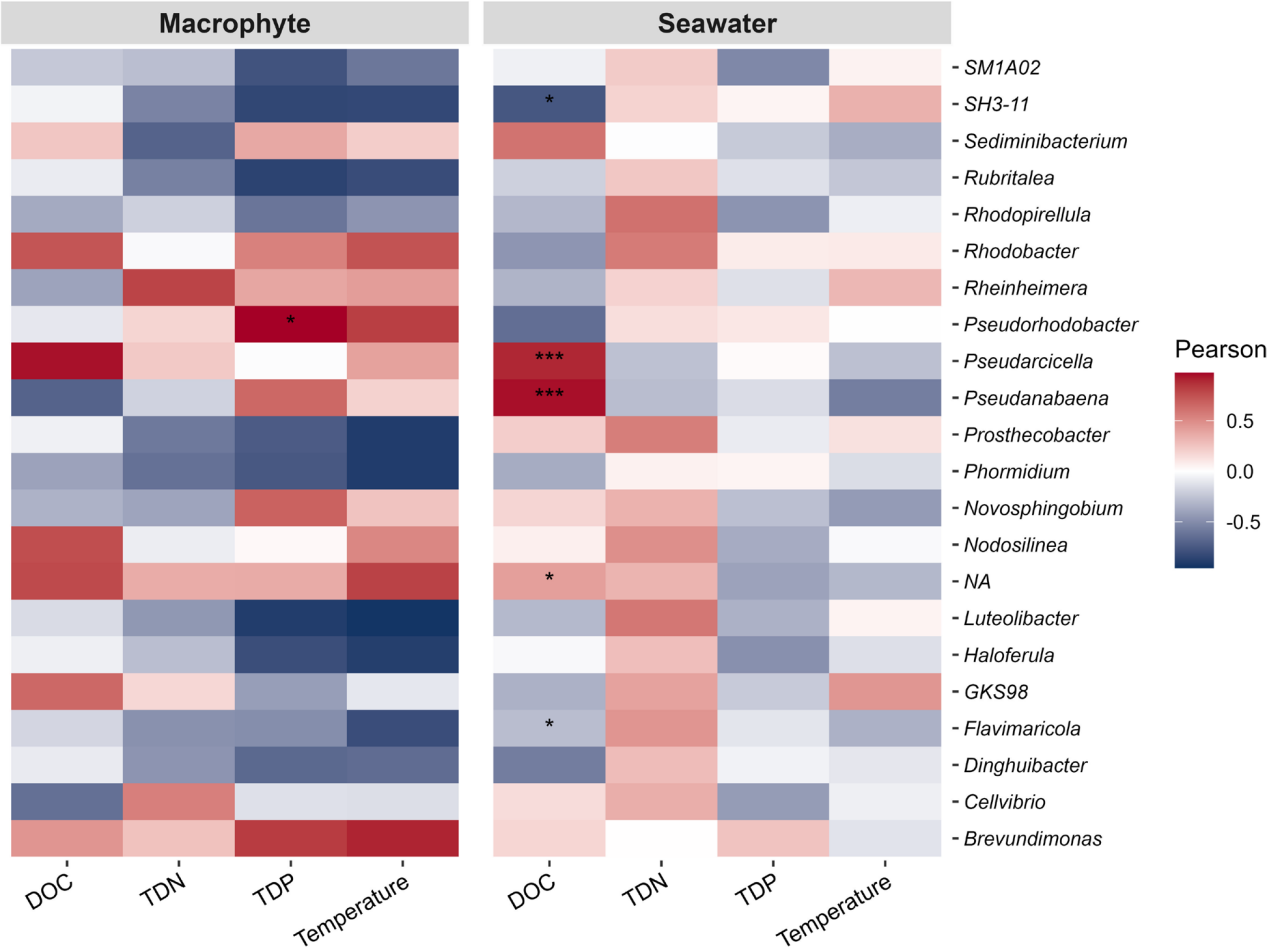
Heat map showing the Pearson correlation analysis between environmental parameters (DOC, TDN, TDP and temperature) and most abundant genus level in seawater samples. False discovery rate (FDR) method was used for the *p* value adjustment. ‘***’ represents highly significant correlation (*p* < 0.0001) and ‘*’ represents significant correlation (*p* < 0.005). NA indicates genera that were not assigned to the genus level.

We compared the abundance of different bacterial genera in seawater and on macrophyte leaves with environmental variables of seawater (Figure 7). Based on significant results of Pearson correlation analysis (*p* < 0.05), *Pseudorhodobacter* on macrophyte leaves showed a positive correlation with TDP. Seawater *Pseudarcicella* and *Pseudanabaena* were positively correlated with DOC, whereas *Flavimaricola* showed a negative correlation to this measured variable.

## 4 Discussion

### 4.1 Distinct Bacterial Communities on Macrophytes and Seawater

Bacterial communities growing on the macrophyte leaves showed marked differences to those that were free‐living in the seawater, as shown in the NMDS plot. Higher Shannon diversity was found on macrophyte samples compared to seawater. The macrophyte leaf surface provides microhabitats for bacterial colonisation and reduced disturbances, which can support diverse bacterial communities on macrophytes. *Proteobacteria* was the most abundant phylum on the macrophytes. Previous studies have shown a high abundance of *Proteobacteria* in various macrophyte species (Whipps et al. 2008; Cúcio et al. 2016; Zhang et al. 2020). A recent study also reported a high abundance of *Proteobacteria* in constructed wetlands with the macrophyte *Vallisneria natans* , promoting the function of the nitrate reduction process (Jiang et al. 2024). *Proteobacteria* have been shown to have a key function as degraders of macrophyte litter (Zhao et al. 2017). This suggested an association between *Proteobacteria* and macrophytes, indicating that they can facilitate the decomposition process in collaboration with other bacterial taxa.

Like for *Proteobacteria*, we observed a significantly higher relative abundance of class *Gammaproteobacteria* in Rundvik Bay. However, the relative abundance did not differ considerably between seawater and macrophytes. This could possible be due to the availability of nutrients or other consistent environmental factors between these sample types (seawater and macrophytes) in Rundvik Bay. Further studies are needed to improve our understanding of the functional potential of *Gammaproteobacteria* on macrophytes rather than focusing merely on taxonomic composition. In both bays, *Cyanobacteriia* showed higher relative abundance on macrophytes. In line with our studies, previous research has shown a direct interaction between cyanobacteria and macrophytes (seagrass) (Hamisi et al. 2009, 2013). The higher abundance of *Cyanobacteriia* on the macrophyte is probably due to the habitat stability for cyanobacteria to thrive in such environments, which promotes growth of cyanobacteria as epiphytes. *Actinobacteria* was one of the significantly higher bacterial classes in seawater samples compared to macrophytes. Members of *Actinobacteria* are specialist bacteria that show high abundance in the Baltic Sea when there is a freshwater inflow (Figueroa et al. 2021), where they may be involved in the degradation of organic matter.

Rhodobacteraceae and Saprospiraceae were the two most abundant bacterial families in the macrophyte samples. The occurrence of Rhodobacteraceae is consistent with previous studies, reporting the presence of this group on the surface of macrophytes (Burke et al. 2011; Mancuso et al. 2016). Saprospiraceae could have been involved in the degradation of various organic compounds, as this group is known to be able to break down complex organic carbon (McIlroy and Nielsen 2014). Likewise, the high abundance of the genera *Pseudorhodobacter*, *Novosphingobium* and *Erythrobacter* on the macrophyte leaves may have facilitated the degradation of complex organic compounds in the surrounding environment, as these bacteria are able to degrade persistent organic compounds, lignin and polycyclic aromatic hydrocarbons (Yao et al. 2024; Linz et al. 2021; Inoue et al. 2005; Maeda et al. 2009). The relatively high abundance of Spirosomaceae in the seawater in Rundvik Bay might be related to the high concentration of DOC in seawater in this bay, although further studies would be needed to confirm such a relationship.

### 4.2 Bacteria Relationships With Environmental Variables

Environmental conditions such as the level of nutrient availability and salinity in particular expose selective pressure for members of bacterial communities. In our study, only a few bacterial genera showed significant relations to environmental variables. One genus of the epiphytic bacteria showed a significant relation to measured physicochemical variables: *Pseudorhodobacter*, which had a positive association with TDP. The TDP concentration ranged from 0.11–0.37 μmol/L, with most of the high values in the Rundvik Bay. *Pseudorhodobacter* is a heterotrophic taxon that has been suggested to be an indicator of remediated water (Yang et al. 2019). Even though the TDP concentration ranged a factor of 3.4, the area can overall be considered relatively undisturbed and not eutrophicated (e.g., Andersson et al. 1996). It is likely that epiphytic bacteria utilise most nutrients from seawater passing by the macrophytes, and since the sea area has been shown to be phosphorus limited (Andersson et al. 1996), it is likely that *Pseudorhodobacter* is one of the bacteria that respond positively to variations in the most limiting nutrient.

For the seawater bacteria, *Pseudarcicella* and *Pseudanabaena* showed a highly significant positive relation to the seawater DOC, while *Flavimaricola* exhibited an opposite relation to this variable. *Pseudarcicella* is a heterotrophic bacterium, which occurs in oligotrophic freshwater systems and has been suggested to be an indicator of good quality lakes (e.g., Guo et al. 2021). The positive relation to DOC indicates that this genus benefited from high carbon availability, but it may also be due to an indirect effect of low salinity, as DOC and salinity are inversely related in the study area.

*Pseudanabaena* is a relatively small filamentous cyanobacterium, which occurs at different salinities and nutrient conditions (e.g., Xiao et al. 2020; Lehman et al. 2021). This genus has been suggested to have a competitive advantage at low nitrogen concentrations (Farnelid et al. 2009; Sun et al. 2023). The observed positive relation of *Pseudanabaena* to DOC might be an indirect effect of low salinity and low nutrient concentrations, as the nutrient concentrations were overall low. Although the observed negative link between *Flavimaricola* and DOC was weak, it might indicate that higher salinity conditions are more optimal for this genus. Even though the above‐mentioned relations would need more comprehensive investigation, we can conclude that environmental variables like nutrient availability, DOC and salinity were influential factors for the occurrence of epiphytic and seawater bacteria.

### 4.3 Metabolic Pathway of Complex Organic Compounds Linked to Epiphytic Communities

The functional capabilities of macrophyte‐associated bacterial communities remain poorly understood. To better understand the functional prediction of bacteria, PICRUSt analysis revealed 14 pathways that were enriched, with significant differences between macrophytes and water samples. Previously, Yu et al. (2022) also identified the functional genes involved in nitrogen metabolism and oxidative phosphorylation of epiphytic bacteria in the submerged macrophyte, *Potamogeton maackianus*. Phosphonate and phosphinate metabolism were significantly higher in macrophyte bacterial communities, which might be due to bacteria often synthesising phosphonates as secondary metabolites containing C—P bonds. In contrast, Sun et al. (2024) found that the pathways involved in phosphonate and phosphinate metabolism were enriched in the surrounding seawater samples of *Zostera marina* . Moreover, an increased abundance of pathways for aromatic compounds degradation was found in the macrophyte samples. This suggests that macrophyte‐associated bacterial communities could influence the degradation of the majority of aromatic ring‐structured compounds (such as nitrobenzoate and catechol). The presence of pathways, such as catechol degradation and aromatic compounds via the beta‐ketoadipate pathway, clearly indicates the functional role of bacteria‐associated macrophytes. Genera such as *Pseudorhodobacter*, *Novosphingobium* and *Erythrobacter* found as epiphytic bacteria may function as degraders of organic and aromatic compounds in the surrounding environment. Future studies are needed to elucidate the functional role of these taxa to better understand their occurrence and interaction with the macrophytes.

In addition, we also identified pathways for carbohydrate degradation, such as starch degradation III and fucose, which could be linked to the degradation of plant‐derived carbohydrates by epiphytes. Further studies are needed to understand the functional interaction of epiphytes in utilising carbohydrates of plant‐derived materials. We found the ECA biosynthesis pathway was abundant only on macrophytes of both bays. One possible explanation for the enrichment of this pathway could be that it plays an essential role in the synthesis of ECA, a component of the outer membrane of *Enterobacterales* (Kuhn et al. 1988), which includes coliform bacteria. In contrast, we found the biosynthesis pathway for UDP‐N‐acetylglucosamine was abundant in seawater samples, which is likely to be involved in the synthesis of peptidoglycan and lipopolysaccharides of the bacterial cell wall (Bouhss et al. 2008). The pathways related to galactose (Leloir pathway) degradation occurred in higher abundance in seawater samples than in macrophyte samples.

The leaves of macrophytes like *P. perfoliatus* are known to contain secondary metabolites, such as phenolic compounds (Smolders et al. 2000). The epiphytic bacteria on the surface of macrophytes can utilise the phenolic substances released by plants as substrates. In our study, we observed the enrichment of certain bacterial genera present on *P. perfoliatus* leaves, suggesting that this macrophyte secretes and releases specific secondary metabolites for shaping the specialist bacterial taxa through symbiotic associations. A previous study by Müller et al. (2007) found that *Myriophyllum spicatum* composed of epiphytic bacteria, like *Matsuebacter* sp., is capable of degrading gallic acid and polyphenols. Therefore, the differences in bacterial communities between planktonic and epiphytic bacteria are most likely due to the plant‐derived metabolites released from *P. perfoliatus* . The macrophyte may secrete specific secondary metabolites based on its requirements, supporting the growth of specific microbial taxa. However, there is still a knowledge gap in the metabolic profiling of *P. perfoliatus* in the subarctic region.

### 4.4 Perfoliate Pondweed Meadows—A Reservoir of Potentially Pathogenic bacteria in the Coastal Northern Baltic Sea

The higher CFU of *Vibrio* and coliforms on the macrophytes from both bays confirmed that the perfoliate pondweed meadows are reservoirs of pathogenic bacteria and possibly function as filters, which sieve the pathogenic bacteria from the seawater, thereby reducing their concentration and the potential risk of harmful diseases (Lamb et al. 2017; Quero et al. 2015). The observed pathogenic bacteria, *Aeromonas* and *Serratia*, are known fish pathogens (Baya et al. 1992; Wahli et al. 2005). They may originate from fish living in the meadows, such as pike, roach, perch and sticklebacks (Snickars et al. 2009).

Coliform bacteria can cause a range of human infections, including gastroenteritis and urinary tract infections (Guentzel 1996). Based on the culture‐dependent plating method, we found some of the cultures of *Serratia* spp., *Vibrio* spp., and *Aeromonas* spp., associated with macrophytes as epiphytes that can otherwise cause diseases in humans, fish and other marine organisms (Baya et al. 1992; Wahli et al. 2005; Frans et al. 2011; Baker‐Austin et al. 2018; Gyraitė et al. 2024). Interestingly, macrophytes had significantly higher abundances of both *Vibrio* and coliform bacteria than the offshore water in Kylören Bay, although not in Rundvik Bay. It may also signify differences in the variations of macrophyte density between the two bays. These pathogenic taxa were, however, not found by the metabarcoding approach, possibly because of their relatively low abundance, which might be difficult to capture using the long‐read sequencing method. In such cases, a culture‐dependent approach can be used as a combination approach to detect the low abundance and novel taxa, especially in the environment we sampled. Nevertheless, metabarcoding analysis also revealed the occurrence of other pathogens. The high abundances of *Legionella* and *Leptospira* in inshore waters in both bays suggest a potential entry point for such pathogens. It is well known that *Legionella* can cause Legionnaires' disease in humans (Muder and Yu 2002) and *Leptospira* can cause *Leptospirosis* (Levett et al. 2001), and *Legionella* has also been linked to humic substances in a recent study conducted in the northern Baltic Sea (Eriksson et al. 2023). Future studies should investigate pathogenicity using biochemical and molecular methods and the actual sources of such potential pathogenic bacteria present on macrophytes and their temporal dynamics. We argue that macrophyte meadows can serve as hot spots for pathogens and may play an important role even for emerging pathogens in the future.

## 5 Conclusion

Even though we only studied two replicated bays, the results were consistent and point to generality, that is, that perfoliate pondweed meadows are a source of potentially pathogenic bacteria and that their loosely attached bacterial communities play a role in the degradation of complex organic carbon compounds that are most likely originating from dead plant materials and humic substances passing by when traversing from land to sea. The macrophyte may synthesise secondary metabolites such as phenolic compounds, supporting the growth of specific epiphytic bacteria and thus favouring a symbiotic association.

Furthermore, we show that macrophyte meadows harbour potentially pathogenic bacteria, such as *Vibrio* and coliform bacteria, and *Legionella* and *Leptospira*. Their occurrences in the meadows may be attributed to inland waters or to juvenile fish and other marine organisms inhabiting the meadows. The results of our study suggest that detection of potentially pathogenic bacteria should be performed by combining traditional plating techniques with metabarcoding, as each method identifies different and important taxonomic groups. The general occurrence of potentially pathogenic bacteria in macrophyte meadows would be considered in light of meadows being indicators of good water quality. Upcoming research may therefore develop a framework for how these bacterial communities can be integrated into monitoring programmes. Further, epiphytic bacteria should be highly relevant in future research focusing on both animal and human health aspects and in pathogen dispersion‐related investigations.

## Author Contributions

**Kesava Priyan Ramasamy:** conceptualization, data curation, methodology, software, formal analysis, investigation, validation, visualization, writing – review and editing, writing – original draft. **Máté Vass:** conceptualization, data curation, methodology, software, formal analysis, validation, visualization, writing – original draft, writing – review and editing. **Johnny Berglund:** conceptualization, methodology, writing – review and editing. **Anniina Saarinen:** methodology, writing – review and editing. **Agneta Andersson:** conceptualization, methodology, investigation, writing – review and editing, writing – original draft, validation, supervision, resources, project administration, formal analysis, funding acquisition, data curation.

## Conflicts of Interest

The authors declare no conflicts of interest.

## Supporting information


**Figure S1:** Concentration of (A) dissolved organic carbon (DOC). (B) Total dissolved nitrogen (TDN) and (C) Total dissolved phosphorus (TDP). Error bars represent the standard deviation value of five samples.
**Figure S2:** The relative abundance of top 10 phyla across all samples and replicates (see sample codes in Table S1).
**Figure S3:** The relative abundance of top 15 families across all samples and replicates.
**Figure S4:** Boxplot showing the alpha diversity indices of seawater (*n* = 5) and macrophytes (*n* = 3). (A) Species richness and (B) Shannon index in Kylören and Rundvik bays. Note that seawater samples consist of inshore, meadow region and offshore samples.
**Figure S5:** Bar plot showing the relative abundance of the KEGG pathway involved in the bacterial metabolism. Error bars represent the significant differences across two groups (macrophyte meadows and water), *p* value < 0.05.

**Table S1:** Coordinates of the sampling locations, physicochemical measurements and CFU counts.
**Table S2:** Filtered quality reads processed using NGSpeciesID pipeline.
**Table S3:** OTU abundance and taxonomy table.
**Table S4:** MetaCyc pathways identified across samples.

## Data Availability

Metabarcoding data were deposited to NCBI SRA database under the accession number: PRJNA922946. 16S rRNA gene sequences from culture isolates have been deposited in GenBank under the accession numbers (OP616840‐OP616850 and OP616944‐OP616971).
