# Supplementary material for: Perfoliate Pondweed Meadows in Northern Coastal Areas—Reservoirs of Diverse Bacteria With Pathogenic Traits and Complex Carbon Metabolism
Source: Environ Microbiol Rep. 2025 Sep 12;17(5):e70180. doi: 10.1111/1758-2229.70180 (PMC12432016; doi:10.1111/1758-2229.70180)
Supplement: Supplementary file 1 — Figure S1: Concentration of (A) dissolved organic carbon (DOC). (B) Total dissolved nitrogen (TDN) and (C) Total dissolved phosphorus (TDP). Error bars represent the standard deviation value of five samples. Figure S2: The relative abundance of top 10 phyla across all samples and replicates (see sample codes in Table S1). Figure S3: The relative abundance of top 15 families across all samples and replicates. Figure S4: Boxplot showing the alpha diversity indices of seawater (n = 5) and macrophytes (n = 3). (A) Species richness and (B) Shannon index in Kylören and Rundvik bays. Note that seawater samples consist of inshore, meadow region and offshore samples. Figure S5: Bar plot showing the relative abundance of the KEGG pathway involved in the bacterial metabolism. Error bars represent the significant differences across two groups (macrophyte meadows and water), p value < 0.05. [file EMI4-17-e70180-s002.pdf]

Supplementary Figures

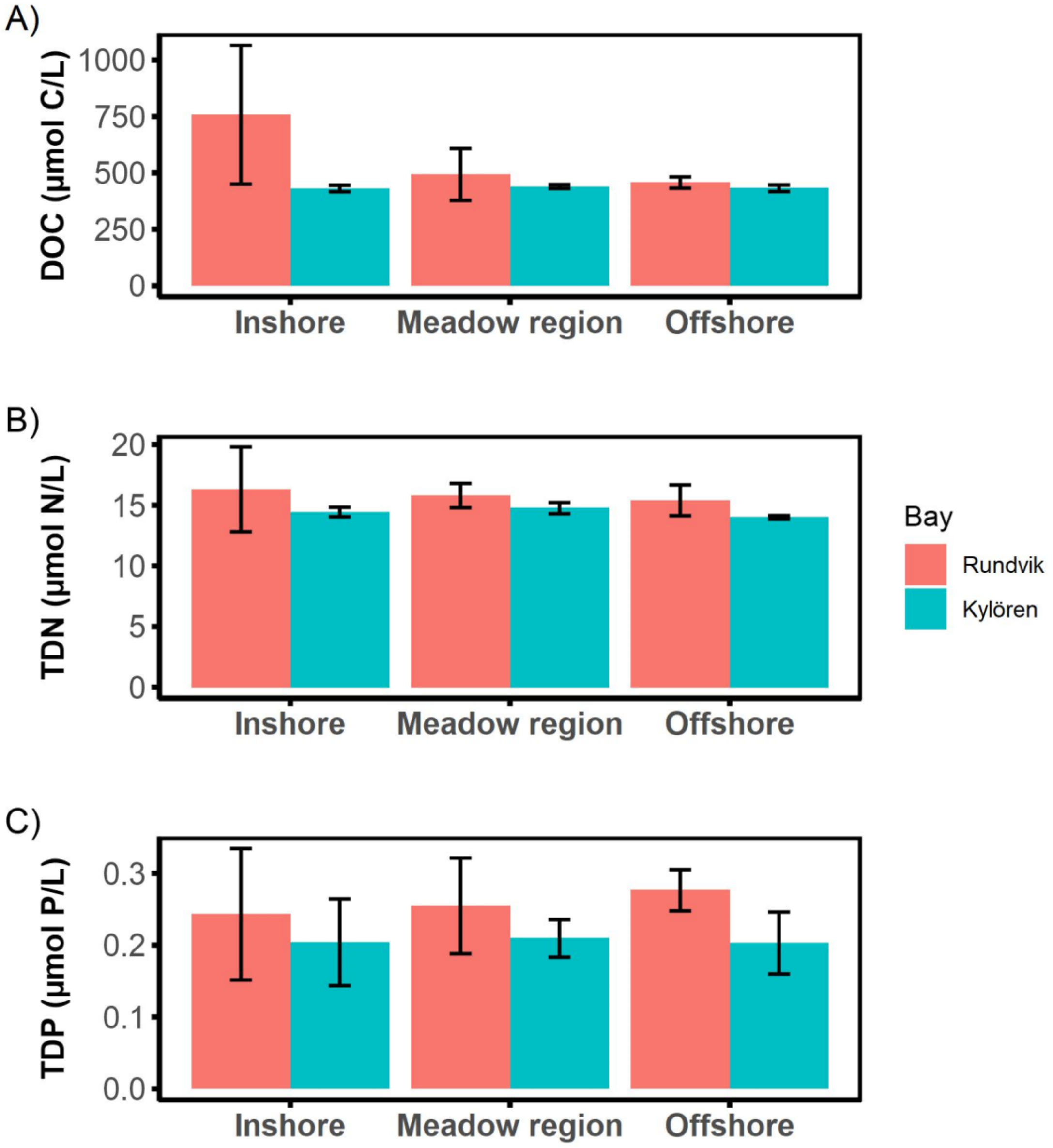

Supplementary Figure 1. Concentration of A) dissolved organic carbon (DOC) B) Total dissolved nitrogen (TDN) and C) Total dissolved phosphorus (TDP). Error bars represent the standard deviation value of five samples.

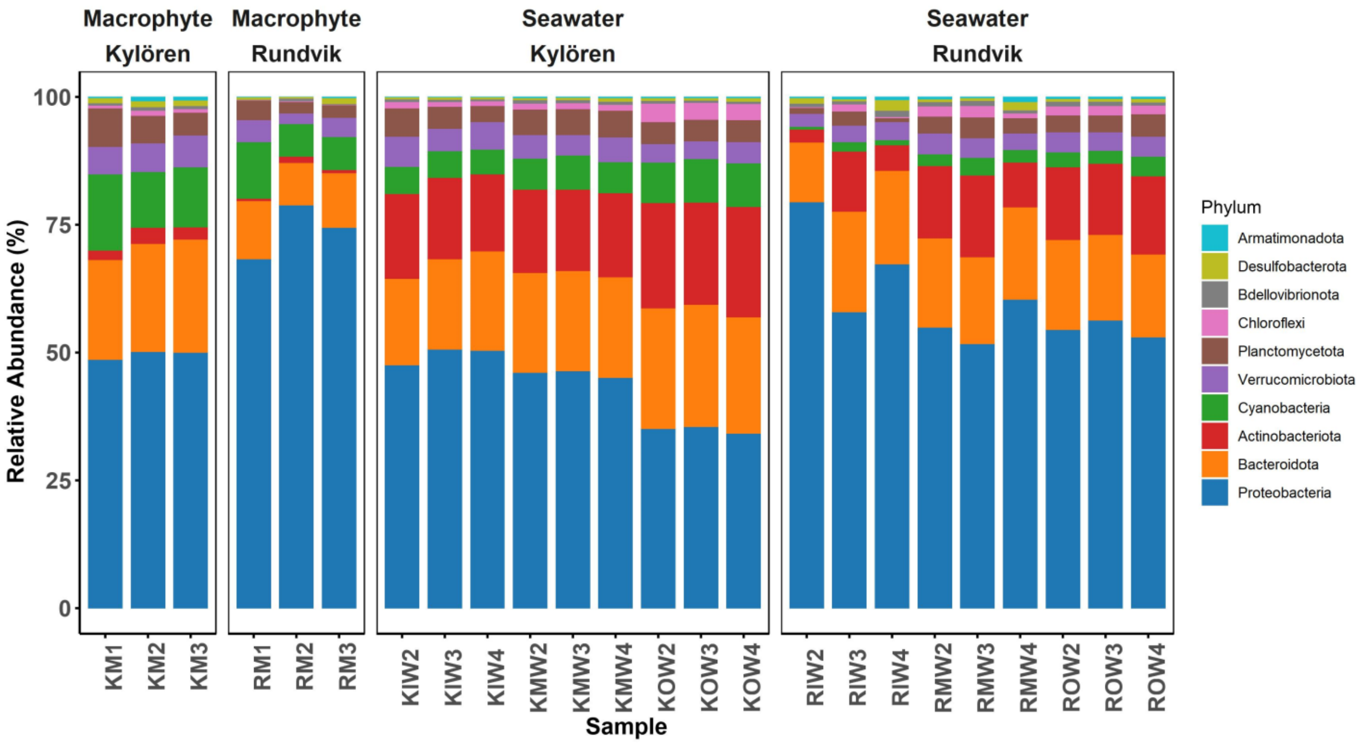

Supplementary Figure. 2 The relative abundance of top 10 phyla across all samples and replicates (see sample codes in Table S1).

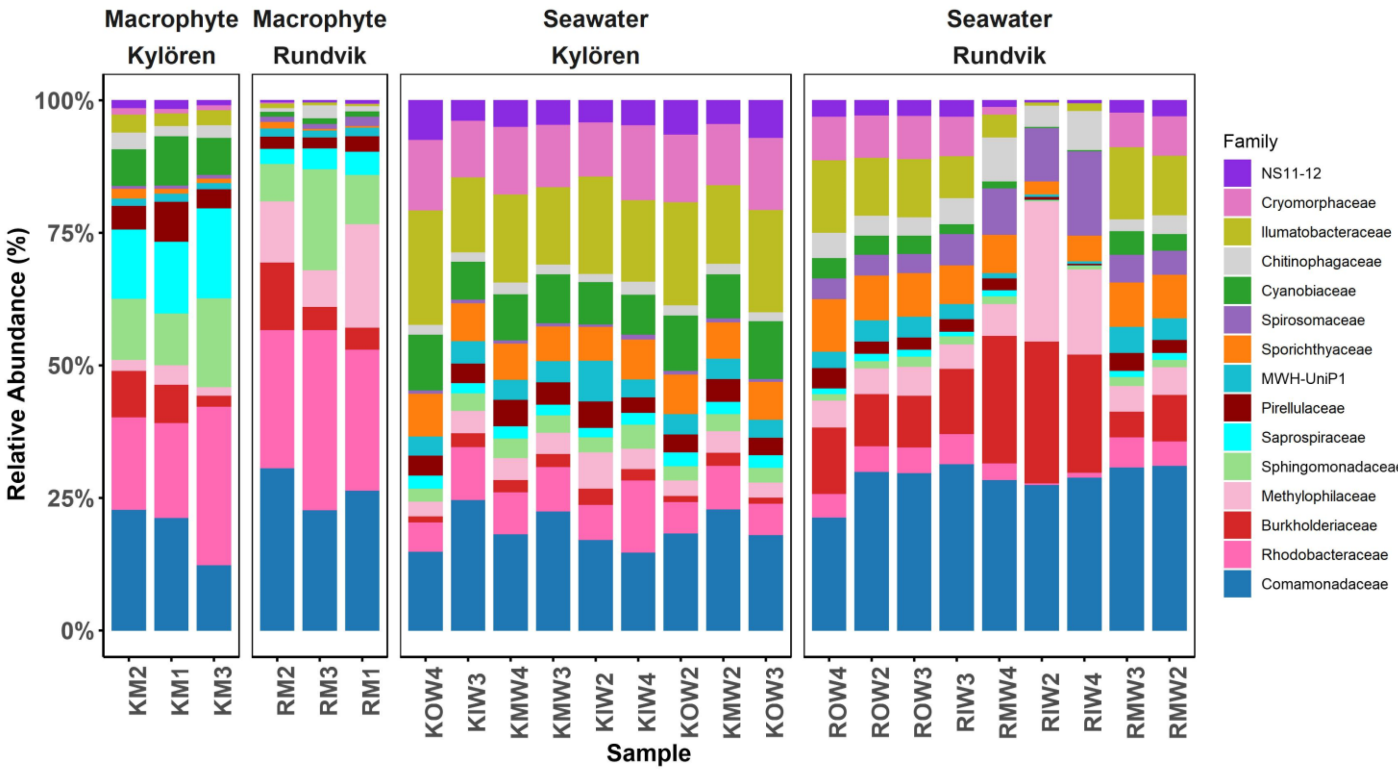

Supplementary Figure 3. The relative abundance of top 15 families across all samples and replicates.

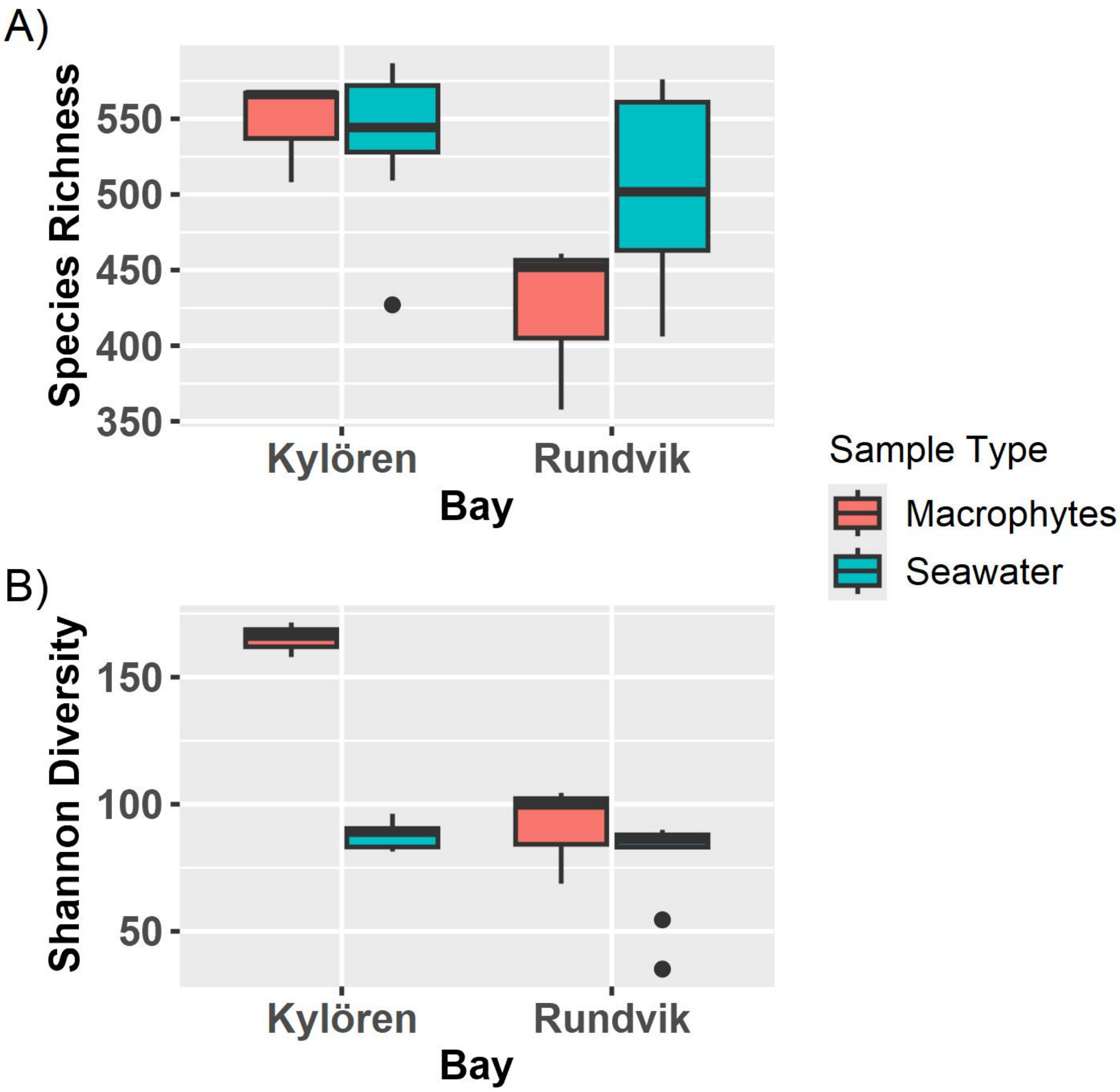

Supplementary Figure 4. Boxplot showing the alpha diversity indices of seawater (n = 5) and macrophytes (n = 3) A) Species richness and B) Shannon index in Kylören and Rundvik bays. Note that seawater samples consist of inshore, meadow region and offshore samples.

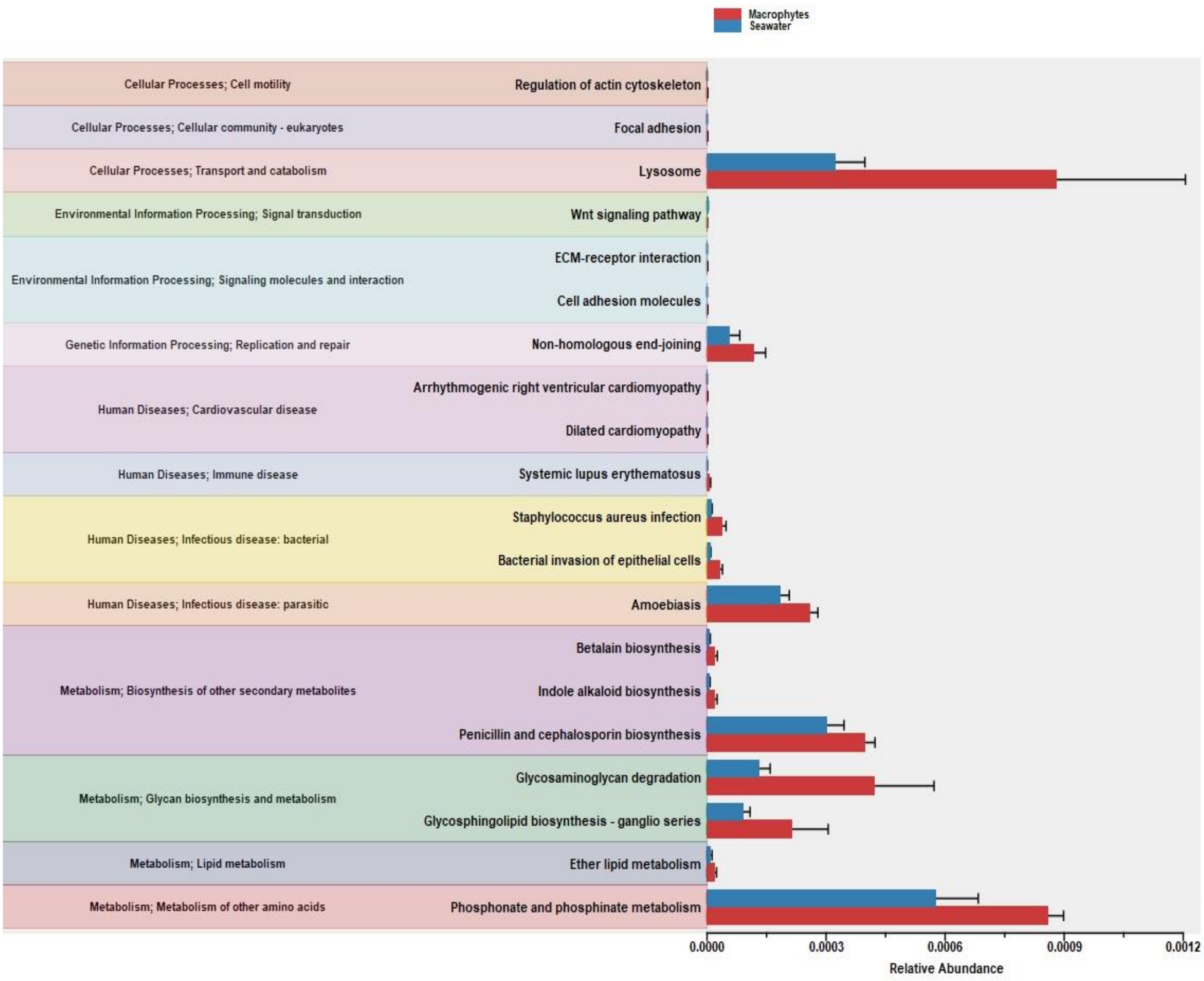

Supplementary Figure 5. Bar plot showing the relative abundance of the KEGG pathway involved in the bacterial metabolism. Error bars represent the significant differences across two groups (macrophyte meadows and water), p value < 0.05.
